# The proportional lack of archaeal pathogens: Do viruses/phages hold the key?

**DOI:** 10.1002/bies.201000091

**Published:** 2011-02-15

**Authors:** Erin E Gill, Fiona S L Brinkman

**Affiliations:** Department of Molecular Biology and Biochemistry, Simon Fraser UniversityBurnaby, British Columbia, Canada

**Keywords:** Archaea, Bacteria, pathogen, phage, virus

## Abstract

Although Archaea inhabit the human body and possess some characteristics of pathogens, there is a notable lack of pathogenic archaeal species identified to date. We hypothesize that the scarcity of disease-causing Archaea is due, in part, to mutually-exclusive phage and virus populations infecting Bacteria and Archaea, coupled with an association of bacterial virulence factors with phages or mobile elements. The ability of bacterial phages to infect Bacteria and then use them as a vehicle to infect eukaryotes may be difficult for archaeal viruses to evolve independently. Differences in extracellular structures between Bacteria and Archaea would make adsorption of bacterial phage particles onto Archaea (i.e. horizontal transfer of virulence) exceedingly hard. If phage and virus populations are indeed exclusive to their respective host Domains, this has important implications for both the evolution of pathogens and approaches to infectious disease control.

## Introduction

There are hundreds of organisms that infect and cause disease in humans, including diverse Bacteria and single-celled Eukarya, as well as a few animals, such as helminths 1, 2. One Domain of life is conspicuously absent from this list: the Archaea. We have yet to find convincing evidence of an archaeal pathogen, although some archaeal genera inhabit the human body and share symbiotic and commensal relationships with a handful of animals and single-celled eukaryotes 3, 4. If archaeal pathogens do exist, it is surprising that of all the archaeal species that have been identified to date 5, none are pathogens (527 archaea classified in the NCBI taxonomy database, or 4,508 if uncultured/unspecified species and strains are included 5). Only a small fraction of Bacteria cause diseases in humans 1, 6. Taylor et al. 6 comprehensively annotated 538 species of bacterial pathogens and the total number of bacterial species in the NCBI taxonomy database is currently 15,919 (151,514 if uncultured/unspecified species are included). Therefore, we estimate that roughly ∼0.36% of bacterial species are pathogenic, including uncultured/unspecified species for a more conservative analysis. Of course, bacterial and archaeal diversities are both larger, but assuming proportions are the same, and if a similar fraction of archaeal species were to cause disease, we would expect to have identified about 16 archaeal pathogens. The absence of pathogenic Archaea in the NCBI taxonomy database is, statistically, highly significant (binomial test *z* = 4.01, *p* < 0.01, or if uncultured/unspecified species are not included: *z* = −4.29, *p* < 0.01). In addition, pathogenic Bacteria are identified by determining the causes of diseases, not by identifying species and then establishing if they are pathogenic. The causative agents for most major infectious diseases have been well characterized, and so identification of archaeal pathogens, if they exist, is more likely given this bias. Several theories have been postulated regarding Archaea and their relationship to disease, which are discussed below, coupled with our proposal for why there is a lack of disease-causing Archaea. Understanding this may be critical to understanding how pathogens evolve and how they may be better controlled.

## Do Archaea possess the ability to cause disease?

As discussed by Cavicchioli et al. 1, there are several characteristics possessed by pathogens that Archaea seem to share. Namely, they are a highly diverse Domain of life that is present in numbers in the environment that would afford them the opportunity to cause disease. They are able to interact with eukaryotic cells in symbiotic relationships, suggesting that pathogenic relationships may be possible. They are present in many animals in large numbers (i.e. human oral cavity, intestine, and vagina) and are recognized by the immune system. Archaea possess “toxin” genes which play roles in the plasmid addiction system 7, but are not secreted by the cell (note that no canonical virulence factors have been yet found in any archaeal species). Archaea are capable of extracellular secretion using the sec and tat pathways. No known archaeal species possesses a complete bacterial type III or type IV secretion system (which are associated with virulence 8); although some components of the type II and type IV apparatuses function in archaeal flagella 1 because of the evolutionary relationship between flagella and these secretory systems. A few genes (such as *tadA*, which encodes a tRNA specific adenosine deaminase 1, 9) that are involved in host interaction can be found in certain Archaea. However, these genes are associated with more general cellular functions and are present in both pathogenic and non-pathogenic Bacteria.

The afore-mentioned characteristics led Cavicchioli et al. to propose that it is likely that there are archaeal pathogens, but that they have not yet been discovered. Since the publication of the Cavicchioli et al. paper in 2003, meta-genomic studies have revealed more about the diversity of microbial life and prevalence of Archaea. Such methods are invaluable tools for elucidating the causes of unknown illnesses. Meta-genomic methods have demonstrated the presence of Archaea in some diseased tissues 10–15, but archaeal pathogens have not been found.

Martin 16 postulates that Archaea are not pathogens because they use different co-factors in their biochemical reactions compared to Eukarya (and Bacteria). Eukaryotes would, therefore, not provide a good source of nutrients for Archaea. However, although Archaea do contain unique co-factors, they still utilize molecules that could be readily supplied by eukaryotic cells, such as amino acids and nucleotides 17. There are also many other benefits to being a pathogen that do not involve using the host as a nutrient source, such as immune evasion and ease of transmission (e.g. the diarrhea caused by Cholera when *Vibrio cholerae* is infected with cholera phage – the diarrhea aids removal of *V. cholerae* from the host bowel back into the environment as part of an elegant interplay between phage and Bacteria 18). While this use of different co-factors may still be relevant, the presence of Archaea in humans indicates that they can live inside a eukaryotic host. Thus, these Archaea are either able to synthesize their own co-factors or utilize co-factors that are obtainable from their environment.

## Could Archaea contribute to disease caused by other organisms?

The presence of methanogenic Archaea has been correlated with various human disease states, such as gum disease, gastrointestinal ailments, and colon cancer 10–15, but no causative relationships have been established. In these situations, Archaea may facilitate the growth of disease-causing organisms rather than causing disease themselves 19. This could be achieved by removing H_2_ from areas where complex microbial communities exist. H_2_ inhibits the growth of some disease-causing Bacteria, and once removed, these species could flourish 19. This hypothesis warrants further examination, yet remains to be confirmed and, of course, is not a case of Archaea being the primary causative agent of disease.

Pathogenic Bacteria may have acquired some of their virulence factors from Archaea through the transfer of “Pathogenic” genes from species that engage in symbiotic or commensal relationships with Eukarya 20, 21. Although lateral gene transfer has been inferred between Bacteria and Archaea 22, the mechanisms that facilitate the transfer have not been characterized, as conjugation appears to differ between the two Domains 23, 24. Studies involving the plasmids of *Sulfolobus* (the only archaeal plasmids to have been investigated thus far) have revealed that this archaeal plasmid transfer process is much simpler than in Bacteria, as only a rudimentary conjugative machinery is encoded on the plasmids (or in any sequenced archaeal genome) 24. In addition, no phages or viruses have been observed that infect both Archaea and Bacteria. Neither Faguy 20 nor Gophna et al. 21 definitively linked pathogenesis with any gene that has been transferred from Archaea to a bacterium. *Escherichia coli* genes identified as having top archaeal BLAST hits (and, therefore, proposed likely to be derived from Archaea) 20 no longer fit this criterion by a more up to date BLAST analysis (data not shown), and the problem of top BLAST hits not necessarily implying cross-Domain horizontal gene transfer has been well described 25. The lack of recognized genes that have been transferred laterally from Archaea to Bacteria may be due to the small number of archaeal genomes sequenced (especially mesophiles that are commensals or symbionts of animals) 21. There are currently only 22 complete methanogen genomes in GenBank, (and 15 known methanogen species that reside in human or animal gastrointestinal tracts 26). An increase in methanogen genome data will likely yield additional information regarding potential laterally transferred genes. However, it remains unclear how such genes might confer pathogenicity to Bacteria but would not to the Archaea from which they were originally derived. Below, we propose an additional hypothesis explaining the lack of archaeal pathogens.

## Hypothesis: Lack of transduction by relevant bacterial phages prevents Archaea from becoming pathogens

Although Archaea possess many traits that are shared by disease-causing organisms and could contribute to diseases caused by other micro-organisms, the fact remains that we have yet to identify a bona fide archaeal pathogen (even though it is, statistically, highly likely that we would have based on sampling to date). The question remains: why do so few (if any) pathogenic Archaea exist?

We propose that the proportional lack of archaeal pathogens is due to mutually exclusive populations of phages and viruses that infect Bacteria and Archaea respectively (or, at least, due to mutually exclusive populations of phages and viruses that include relevant, infectious disease-associated bacteriophages), coupled with the association of bacterial virulence with phage/mobile elements.

## Phages: The source of many pathogen-associated genes

One of the changes that occurs in a benign bacterial strain that enables it to become a pathogen is the acquisition of novel genetic material via horizontal gene transfer 27. Mobile genetic elements, including phages, play a key role in this gene transfer process and in generating bacterial genomic diversity 28. Many genes that enable Bacteria to become pathogenic are clustered in or adjacent to such elements. In fact, a study of 631 genomes recently showed that virulence factors, as well as pathogen-associated genes, are disproportionately associated with genomic islands (an inclusive grouping of larger mobile genetic elements, including phages), with high statistical significance 8, 29. Most genomic islands are derived from phages 8, 30–32. Since such studies tend to under-predict the occurrence of genomic islands and phages, the association of virulence genes with genomic islands and phages is likely to be even higher 8, 30. There are many individual studies of pathogens that support this observation and the related role of phages in virulence, for example the integration of phages has been shown to help *Staphylococcus aureus* adapt to its human host during infection 33. It is logical that virulence factors would be associated with phages and related mobile elements such as genomic islands 8. Otherwise, such virulence factors would simply become extinct if the bacterium they were contained in became too virulent and killed its host. Virulence factors are better able to survive through their association with the more flexible phage gene pool, through which genes can transfer in and out of bacterial genomes.

Extensive lists of genes in Bacteria, which are associated with virulence and are derived from phages, have been compiled 34, 35. From our analysis, the great majority of these virulence genes are from phages belonging to the families myoviridae (phages with long contractile tails) and syphoviridae (phages with long non-contractile tails), plus a few others including inoviruses and podoviruses. However, this could reflect a bias in the available phage genome data, as the vast majority of sequenced phages are siphoviruses and myoviruses 35. Members of these phage families infect both Gram-positive and Gram-negative Bacteria 35 and so comparing these phages with archaeal viruses is, perhaps, most relevant.

## Archaeal viruses differ from bacterial phages in many respects

The large extent of global viral and phage diversity is slowly coming to light, especially due to recent meta-genomic studies. For instance, Angly et al. 36 estimate that several hundred thousand viral species inhabit the world's oceans (vs. ∼151,000 known bacterial species). It is certain that our databases hardly reflect this amazing diversity in terms of viral and phage genome data. Nevertheless, studies of archaeal viruses seem to indicate that their genomes are part of a gene pool that is separate from phages that infect Bacteria. Viruses that infect members of the Crenarchaeota (one of the primary archaeal phyla) have morphologies that are distinct from those found in bacterial phages or eukaryotic viruses, and their sequences rarely match anything in Genbank from other phyla (according to a BLAST analysis with default cut-offs), including bacterial and bacteriophage sequences 37, 38 (updated BLAST analysis data not shown). In addition, some viruses that infect members of the Euryarchaeota (another primary archaeal phylum) do not have similarities to any bacteriophages (morphologically) nor to any sequences in Genbank 38.

Though these archaeal viruses are clearly different from bacterial phages, there are some archaeal viruses associated with members of the Euryarchaeota that belong to the myovirus and siphovirus families. Since myoviruses and siphoviruses can infect Bacteria, and some mesophilic members of the Euryarchaeota can colonize humans and other animals, it is these families that need to be examined more closely to identify potential similarities between archaeal viruses and bacterial phages. Taxonomic assignment to these families is based on morphological similarity, protein sequence similarity, and genome architecture. However, only three archaeal myoviruses and siphoviruses have been sequenced (reviewed in ref. 38): Phi Ch1 (phiH-like myovirus), psiM1 (psiM1-like siphovirus) and psiM100 (psiM1-like siphovirus). All of the described myoviruses that infect members of the Euryarchaeota belong to the phiH-like genus, and the siphoviruses belong to the psiM1-like genus. These “genera” exclusively comprise viruses that infect Archaea 38. In fact, to date, there are no reports of any archaeal virus that can infect Bacteria, nor any report of a bacterial phage that can infect Archaea. However, since the sample size of thorough investigations of this topic is so low, we investigated further on a gene phylogeny level what is known about archaeal virus and bacterial phage evolution (particularly in the myovirus and siphovirus families found in both Archaea and Bacteria).

## Gene-based phylogeny of archaeal viruses and bacterial phages – no evidence of recent gene exchange

Because of the mosaic nature of phage and virus genomes (they evolve by frequently exchanging genome modules), plus their use of host machinery for some essential functions, there is no common gene that is present in all myoviruses and siphoviruses that infect Bacteria 39. Rohwer and Edwards 40 built a phylogenetic tree of all double stranded DNA bacteriophages and archaeal viruses based on the presence/absence of orthologous genes in their genomes. Glazko et al. 41 used a similar methodology, but also incorporated the similarity of orthologous genes into their tree building method. Interestingly, both groups found that siphoviruses are not monophyletic. Rohwer and Edwards 40 found that archaeal viruses (psiM100 and psiM2) together form a long branch, to the exclusion of all other phages, suggesting that there have not been recent interactions between these archaeal viruses and bacterial phages, but rather that they have an ancient shared ancestry. Glazko et al. 41 also found that archaeal viruses branched together to the exclusion of bacterial phages. There are 18 clades that consistently branch together (the archaeal viruses being one of them), but the relationships between clades do not appear to be well resolved.

We examined sequenced archaeal viruses in order to determine what their genes are related to. Phylogenies were constructed for 84 genes from PsiM100, PhiCh1, and HF1 (which is alternatively classified as a halovirus and a myovirus 38) and there were no cases where lateral transfer from a bacterium or bacterial phage could be exclusively inferred. Several authors have suggested that there is a common gene pool for all tailed bacteriophages and archaeal viruses (myoviruses and siphoviruses) 38, 39, 42. Although it is believed that tailed archaeal viruses and bacterial phages share a common, ancient ancestry, there is no evidence that they have interacted recently with each other but, rather, most likely form their own clade.

In summary, archaeal viruses are either grossly different in morphology and function from bacterial phages, or they appear to form their own clade, separate from anciently-related bacteriophages of the same morphological family (tailed phages). There is no evidence of archaeal virus infection in Bacteria or vice versa. In addition to this phylogeny-based analysis, we also examined the receptors to which bacterial phages and archaeal viruses bind, to investigate whether or not bacteriophages could infect Archaea.

## Bacteriophage receptors in Bacteria, and their structures, are largely absent from Archaea

**Figure 1:**
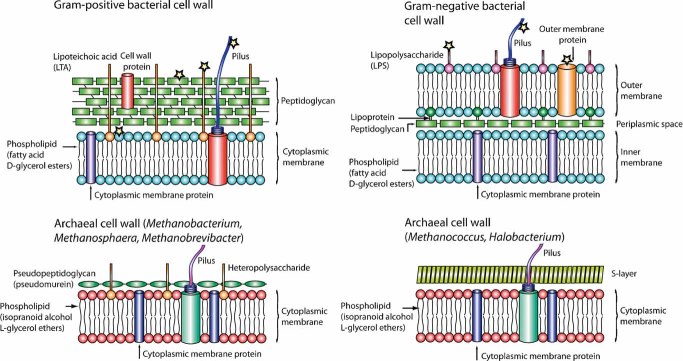
Phage/virus receptor locations in Gram-positive Bacteria, Gram-negative Bacteria, and two types of Archaea. Cell wall and membrane structures are depicted for select classic groups of Bacteria and Archaea (i.e. mesophilic Archaea noted for colonizing humans). Known phage/virus receptor sites are indicated by stars. Note that most of the components to which bacterial phages attach (peptidoglycans, lipopolysaccharides, lipoteichoic acid, and fatty acid D-glycerol ester phospholipids) are absent in Archaea. Although both Bacteria and Archaea possess membrane proteins and pili, they are not highly conserved between these Domains of life.

The ranges of hosts for phages are diverse, from single host strains to over 20 strains and different species 43–45. Phages recognize their hosts via extra-cellular receptors that can be located in the outer membrane of Gram-negative Bacteria, in the cell wall of Gram-positive Bacteria or associated with the flagella or the pili of both Gram-positive and Gram-negative species 46 (Fig. 1). The mycobacterial phages that have been characterized bind to glycolipids that are attached to the outside of peptidoglycan and arabinogalactan cell wall 47. The portion of each phage that recognizes and binds to a receptor is also highly variable, even among phages that have similar receptors on their hosts 46. Receptors for archaeal viruses have not been well characterized. The cell walls and membranes of Archaea are very different from those of Bacteria; their lipids are ether-linked, sugars are joined by different chemical bonds and their amino acids are l- rather than D-isomers 48. In addition, their cell walls can be made of polysaccharides, proteins, or pseudopeptidoglycans 49, and all characterized archaeal pili are structurally divergent from those found in Bacteria 50 (although few archaeal pili have been described). Archaeal flagella also appear to have a different ancestry, and possess many divergent characteristics (such as N-linked glycans vs. O-linked glycans, and a different mode of assembly), from bacterial flagella 51. Therefore, the structures that bacterial phages recognize and bind to are largely absent from archaeal cells (Fig. 1). Given that these components are required for phage infection, it seems unlikely that bacterial phages would be able to infect archaeal cells.

It is important to note, however, that phage anti-receptors (the portions of phages that bind to receptors on bacterial cells) can be modified to change the host range of a phage or the receptor that the phage binds to 43. It is unknown whether anti-receptor mutations would allow phages to bind to an archaeal receptor, but such mutations might also abolish the ability of phages to bind to their natural hosts. It is equally possible that bacterial phages are incapable of transferring their DNA to archaeal cells, even if attachment of a phage were to take place, because of intrinsic differences in DNA processing and/or translocation machinery.

## The archaeal virus and bacterial phage gene pools are likely independent

Based on our analyses, and the reports of others, there appears to be no strong evidence of gene exchange between bacteriophages and archaeal viruses, no evidence that bacteriophages would be able to bind to Archaea due to a lack of suitable receptors, and no evidence in general of any bacteriophages being able to infect Archaea. Without access to the bacterial phage gene pool and its associated virulence genes, Archaea have reduced opportunities to obtain virulence genes. The same holds true for access by Archaea to viruses that infect eukaryotic pathogens, which are even more different. Although Archaea do contain genes that could facilitate host interactions, they lack any canonical virulence factors, including the type III and type IV secretion systems and toxins that were recently found to be strongly associated with virulence in a more systematic study that complemented anecdotal observations 8. If indeed there is extremely limited genetic exchange between bacterial and archaeal viruses, and virulence factors such as type III secretion systems and toxins highly associated with bacterial pathogens cannot be easily created through convergent evolution 8, this may explain, at least in part, why a bona fide archaeal pathogen has yet to be discovered. Again, this may be true for the relationship between eukaryotic pathogens and their viruses. In essence, virulence probably developed early and independently in the bacterial and eukaryotic Domains of life, creating virulence mechanisms that were not easily replicated by convergent evolution in the Archaea. The rarity of genetic exchange between Bacteria, Archaea and Eukarya, and in particular the lack of exchange of their associated phages/viruses/mobile elements, helped to ensure that these Domains of life remained separated to an identifiable degree, whilst also ensuring that virulence factors from Bacteria and Eukarya did not transfer to Archaea, thereby contributing to the lack of development of archaeal pathogens.

## Why have Archaea not developed virulence independently of Bacteria?

Although Archaea do not appear to have access to the bacteriophage gene pool containing bacterial virulence factors, this does not explain why Archaea have not developed their own mechanisms of virulence, independently of Bacteria. The evolution of virulence seems to be a relatively rare event. However, this fact alone does not explain why Archaea have failed to become pathogenic. The answer may lie in the manner in which Bacteria become pathogenic. There is substantial evidence of phages converting benign, environmental Bacteria into disease-causing organisms (see refs. 35, 52 for reviews). In general, it is phages that drive the transition into a pathogenic lifestyle and, therefore, confer the ability to infect eukaryotes. Thus, Bacteria are simply a vehicle to allow phages to infect eukaryotes. Therefore, eukaryotic viruses infect eukaryotes, and bacteriophages transduce Bacteria, which allows them to infect Eukarya. This is a complex arrangement (i.e. phages evolving genes that facilitate infection of the host of their bacterial host), and would not be a trivial one-step development in evolutionary terms. In fact, it would likely involve a multi-step process that may not be easily duplicated.

We would like to point out that the transition from a free-living to a pathogenic lifestyle is a complex process, which is influenced by many genetic and environmental factors. The hypothesis we describe here concerning Archaea and their viruses is meant to clarify certain generalities of this complex process, rather than to encompass the vast range of factors that could contribute to an organism becoming pathogenic.

## Toward a better understanding of pathogen evolution

It is important to appreciate that there are pathogens yet to be discovered. Whether there are any Archaea among these pathogens remains to be seen. However, it is possible that no definitive archaeal pathogens will be uncovered. We hypothesize that virulence gene-encoding bacteriophages cannot interact with Archaea, thereby hindering the ability of Archaea to become pathogens. It is possible that other aspects of the interplay between Archaea and their viruses may not be appropriately balanced (e.g. more lytic viruses) to allow virulence factors to be maintained in an archaeal mobile gene pool. In addition, other intrinsic features of Archaea may play a role, such as their use of different co-factors. In order to test our hypothesis, as well as to test the hypotheses proposed by others, it is vital to gain more information about Archaea that inhabit the human body and other animals. Archaeal interactions with their hosts, Bacteria, and bacteriophages, all sharing the same niche should be characterized. In addition, very little is known about the viruses that infect archaeal species. Further study of the mechanisms of bacteriophage-Bacteria and archaeal virus-Archaea interactions will help elucidate the likelihood of cross-Domain gene transfer among these species and shed light on their potential to receive genes that would confer pathogenicity.

## Implications and conclusions

We know that virulence-associated genes in Bacteria are highly associated with phages, probably by selection, so that these eukaryote host-killing genes can more flexibly survive by transferring in and out of Bacteria and be maintained in the bacteriophage gene pool. Based on the data discussed above, it is unlikely that a bacterial phage would be able to transduce an archaeal cell. So, if key virulence factors such as the type III secretion systems and pathogen-associated toxins mentioned above are difficult to replicate through convergent evolution, the lack of gene exchange from bacteriophages to Archaea may explain why so few (if any) archaeal pathogens exist.

This hypothesis, and the lack of archaeal pathogens in general, has broader implications for the evolution of the domains of life (i.e. supporting the rarity of cross-domain gene transfer) and implications even for approaches to infectious disease control. It further emphasizes the point that the evolution of virulence is not something that is easily “re-invented”, but rather that gene exchange from pathogens to non-pathogens, via a mobile gene pool, is critical to the development of virulence. The interplay between Bacteria and phages, as seen, for example, in the elegant studies of Cholera, is vital to ensuring that some pathogens can exist 18. Infectious disease control measures need to better incorporate an understanding of the important role phages play in infectious disease evolution and, on a shorter time scale, the important role they may play in infectious disease outbreaks. Simply killing off a bacterial pathogen with an anti-microbial agent does not address the true source of virulence, i.e. those phages that disproportionately harbor virulence genes by selection. Approaches for infectious disease control need to better target the actual virulence genes causing disease, such as through anti-infective (anti-virulence) drugs, instead of targeting the bacterial carrier of these genes through anti-microbials. Ironically, through a better understanding of non-pathogenic Archaea, we may better understand pathogens, and may develop infectious disease control measures that truly appreciate how virulence evolved and is maintained.
